# Construction of nomogram for predicting pulmonary hypertension on echocardiography in chronic obstructive pulmonary disease: a single-center retrospective study

**DOI:** 10.1515/med-2026-1475

**Published:** 2026-07-20

**Authors:** Aixia Huang, Tianyu Pan, Yang Gao, Guowen Ji, Ruihong Song, Wenjuan Liu

**Affiliations:** Department of Laboratory Medicine, Respiratory and Critical Care Medicine, Fengxian District Central Hospital, Shanghai, China

**Keywords:** chronic obstructive pulmonary disease, pulmonary hypertension, nomogram, restricted cubic spline analysis

## Abstract

**Objectives:**

To identify independent predictors of pulmonary hypertension (PH) on echocardiography in chronic obstructive pulmonary disease (COPD) patients during acute exacerbations.

**Methods:**

We retrospectively enrolled 151 patients hospitalized for acute COPD exacerbation. Patients were divided by echocardiography into COPD with PH (n=75) and COPD without PH (n=76) groups. LASSO regression screened 17 laboratory indicators to identify the optimal variable subset. Multivariate logistic regression determined independent PH predictors, and a nomogram was constructed. The discrimination and calibration of model were assessed.

**Results:**

LASSO regression analysis screened out 6 optimal variables (age, albumin, cystatin C, red blood cell distribution width (RDW), pH, and CD4/CD8 ratio). Multivariate logistic regression analysis revealed that serum albumin, cystatin C, RDW, pH, and age were independent predictors of PH. The nomogram constructed using these six variables (age, serum albumin, cystatin C, RDW, CD4/CD8 ratio, and pH) demonstrated a C-index of 0.800 (95 % CI: 0.731–0.869). The calibration curve showed that the predicted probability of the model was in good agreement with the actual observed values.

**Conclusions:**

The nomogram constructed based on 6 variables has good predictive efficacy for PH on echocardiography in COPD patients during acute exacerbations. It can provide a convenient risk stratification tool for clinical practice.

## Introduction

Chronic obstructive pulmonary disease (COPD) is a common respiratory condition characterized by irreversible and progressive airway restriction and clinical symptoms of cough and dyspnea. Epidemiological research has suggested that COPD is emerging as a global public health concern, causing significant morbidity, mortality and enormous economic burden [1]. Pulmonary hypertension (PH) is a well-recognized, serious, and potentially fatal long-term complication of COPD, closely associated with right ventricular dysfunction. PH due to COPD (PH-COPD) is classified as pre-capillary PH (WHO Group 3) and correlates with markedly reduced functional capacity, quality of life, and survival [2]. Research demonstrates that patients with PH-COPD suffered worse clinical courses with more frequent exacerbation episodes and emergency visits, greater needs of healthcare resources, and decreased survival [3]. Early identification and appropriate interventions, such as long-term supplemental oxygen therapy, have been documented to improve clinical symptoms and may improve patient survival [4].

PH-COPD in the early stages is typically asymptomatic that defies early detection and treatment [5]. While right-heart catheterization remains the gold standard for measuring pulmonary artery pressure and pulmonary arterial wedge pressure, it is invasive and requires specialized equipment and expertise. Echocardiography offers a non-invasive and convenient alternative for estimating systolic pulmonary arterial pressure as a surrogate diagnostic marker for PH [6]. In addition, echocardiographic measurements of right ventricular function, such as tricuspid annular plane systolic excursion, right ventricular free-wall strain, and tricuspid annular velocity, would also provide insight into the possibility and severity of PH [7]. Nonetheless, echocardiography is also limited by operator experience, and lung hyperinflation associated with COPD also renders echocardiographic assessment technically difficult in this population. Importantly, the 2022 ESC/ERS Guidelines classify TTE as a probability estimation tool, with right heart catheterization required for definitive diagnosis [2], and diagnostic accuracy declines specifically in COPD subgroups [8].

Therefore, early and accurate identification of patients with PH-COPD by clinical parameters and routinely performed laboratory tests would be particularly helpful in resource-limited settings. To the best of our knowledge, there are no studies reporting the application of easily obtainable parameters for the prediction of PH in COPD patients. Thus, the aim of this study is to identify independent predictors of PH during acute exacerbations and to construct a corresponding diagnostic nomogram to facilitate clinical management.

## Methods

### Study population

Patients hospitalized for acute exacerbation of COPD at Shanghai Fengxian District Central Hospital between March 2017 and March 2018 were retrospectively identified and included. COPD diagnosis was based on clinical symptoms of productive cough and dyspnea, along with lung function tests showing a forced expiratory volume in one second (FEV1) to forced vital capacity (FVC) ratio <0.7, according to the 2017 Global Initiative for Chronic Obstructive Lung Disease (GOLD) criteria [9], 10]. Inclusion criteria comprised treatment-naive patients. Exclusion criteria were: 1) Patients with congenital heart disease or infectious valvulopathy; 2) Patients with PH secondary to non-COPD etiologies, including primary pulmonary arterial hypertension (PAH, WHO Group 1) or secondary PH due to pulmonary embolism, left heart disease, or other causes; 3) Patients with lung malignancy, bronchiectasis, asthma, or interstitial pulmonary fibrosis; 4) Portal hypertension; 5) Connective tissue diseases involving the lungs; 6) Hepatorenal insufficiency, defined as serum creatinine exceeding sex-specific normal ranges or aminotransferase levels >2 times the upper limit of normal; 7) Patients with sleep apnea syndrome; 8) Patients with acute infections; 9) Patients who underwent surgery within the preceding 3 months; 10) Patients exhibiting a bronchodilator response to exclude asthma or asthma-COPD overlap syndrome. The study protocol was approved by the Ethics Committee of Shanghai Fengxian District Central Hospital (NO. 2021-KY-27). Informed consent was waived due to the retrospective nature of the study.

### Data collection

We collected relevant data from the electronic medical records. Patients were classified into A, B, E groups based on the severity of symptoms and the risk of exacerbation according to the GOLD Guidelines (2023 edition). Specifically, the following data at admission were retrieved, including patient’s age, sex, ABE group, laboratory test results and arterial blood. The laboratory parameters included white blood cell count, eosinophil count, hemoglobin level, red blood cell distribution width (RDW), serum albumin, cystatin C, D-dimer, fibrinogen, high-sensitivity C-reactive protein, procalcitonin, and B-type natriuretic peptide. Data from arterial blood gas analysis, including oxygenation saturation rate, lactic acid, pH, oxygen pressure and carbon dioxide pressure. Pulmonary function test results were obtained during a clinically stable period following admission. Patient’s immune status was analyzed with flow cytometry to measure CD3 ^+^, CD4 ^+^ and CD8 ^+^ T cell count.

### Measurements

The MasterScreen PFT System (Jaeger, Baglia, Germany) was used to measure patient’s pulmonary function. The same pulmonary function test dataset was used both to confirm COPD diagnosis (post-bronchodilator FEV1/FVC ratio<0.70) and for subsequent statistical analysis. Parameters including FVC and FEV1 were assessed, with data expressed as the percentage of FEV1 relative to the predicted value. Echocardiography was performed during the stable phase of hospitalization by an experienced sonographer using a GE LOGIQ-9 ultrasound system. PH was defined as an estimated pulmonary artery systolic pressure≥40 mmHg [11], [12], [13]. Previous studies have demonstrated that echocardiographic estimates of pulmonary artery systolic pressure exhibit a sensitivity of 43–90 % and a specificity of 60–83 % for PH diagnosis [14], [15], [16].

### Statistical analysis

Data were presented as mean ± standard deviation or median (interquartile range) for continuous variables, as appropriate, with normality assessed using the Shapiro-Wilk test. Comparisons between COPD patients with and without PH were enabled by Student’s t-test and Mann-Whitney *U* test for variables with and without normal distribution, respectively. Spearman’s correlation analyses were performed to assess correlations between albumin, cystatin C, white blood cell count, eosinophils, and other parameters with FEV1 % predicted and FEV1/FVC ratio. Least absolute shrinkage and selection operator (Lasso) regression was applied to exclude collinear variables and obtain the minimal variable set for optimal model fitting. Univariate and multivariate logistic regression analyses were then conducted to evaluate the association between independent variables and PH in COPD. A nomogram to predict PH in COPD patients was then established. The discrimination and calibration of the nomogram were evaluated by C-index and calibration curve, respectively, and the diagnostic value of the model was assessed using the receiver operating characteristic (ROC) curve. Statistical analysis was performed by SPSS 24.0 software (IBM, Armonk, New York) and R software v. 4.2.2. A two-sided p<0.05 indicates statistical significance.

## Results

### Study population

**Figure 1:**
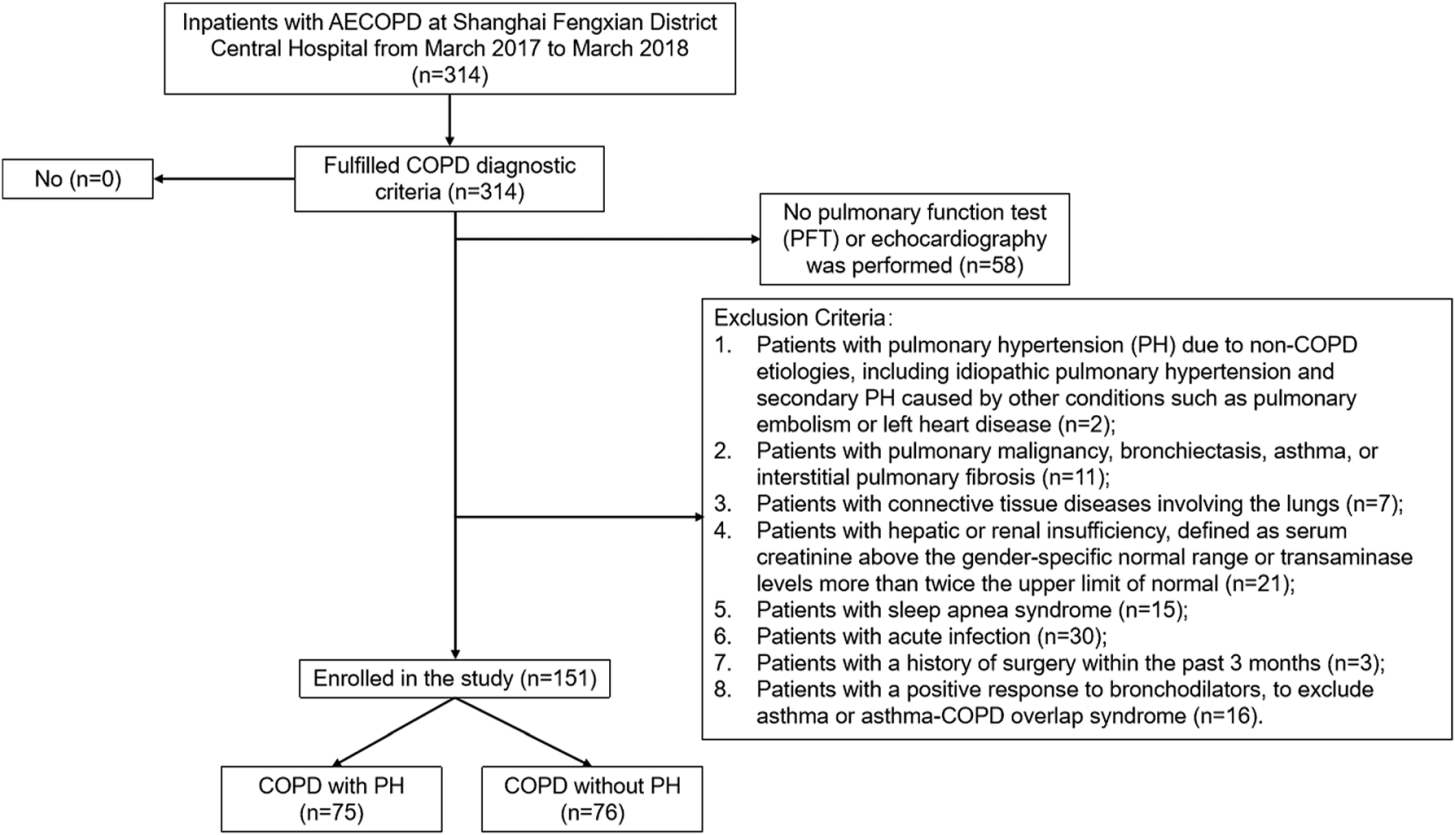
Flow diagram of patient enrolment and study design.

From March 2017 to March 2018, 314 patients with COPD were enrolled in our hospital (Figure 1). After excluding 58 patients who lacked pulmonary function test or echocardiographic data and 105 patients who did not meet the inclusion criteria, a total of 151 patients were included in the final analysis. According to echocardiographic findings, the study population was stratified into two groups: COPD with PH (n=75) and COPD without PH (n=76). As shown in Table 1, compared to those without PH, COPD with PH patients were associated with significantly lower pH, and significantly higher serum albumin, cystatin C and RDW (all p<0.05).

**Table 1:** Comparison of baseline characteristics between COPD patients with and without pulmonary hypertension (PH) 1.

Variables	Without PH (n=76)	With PH (n=75)	p-Value
Age, years	78.5 (70.0, 86.8)	79.0 (74.00, 83.0)	0.938
Albumin, g/L^a^	31.69 ± 5.11	34.68 ± 4.93	<0.001
Cystatin C, mg/L	1.70 (1.36, 2.01)	2.15 (1.50, 3.60)	<0.001
WBC count ( × 10^9^/L)	6.80 (5.20, 10.16)	7.64 (5.57, 10.07)	0.404
Eosinophil count ( × 10^9^/L)	0.05 (0.01, 0.13)	0.02 (0.00, 0.10)	0.115
Hemoglobin, g/L^a^	114.61 ± 26.99	121.33 ± 24.42	0.110
RDW, %	47.6 (44.6, 53.3)	50.2 (46.9, 59.8)	<0.001
Hs-CRP, mg/L	24.8 (6.0, 65.8)	12.8 (3.7, 45.1)	0.267
Fibrinogen, g/L	3.13 (2.67, 4.55)	3.17 (2.38, 4.32)	0.508
D-dimer, mg/L	1.4 (0.9, 2.9)	1.5 (0.8, 3.1)	0.851
CD3, %	57.12 (46.31, 67.52)	60.09 (53.10, 63.22)	0.179
CD4, %	29.19 (23.20, 31.53)	30.14 (22.88, 37.34)	0.076
CD8, %	24.63 (17.73, 33.91)	26.52 (16.81, 33.14)	0.760
CD4/CD8	1.12 (0.78, 1.65)	1.09 (0.85, 2.01)	0.498
Procalcitonin, ng/ml	0.10 (0.10, 0.15)	0.10 (0.10, 0.18)	0.587
BNP, pg/ml	261.3 (123.0, 659.0)	247.2 (85.0, 1,184.6)	0.842
pH	7.40 (7.35, 7.45)	7.38 (7.34, 7.41)	0.016
ABE group^b^			<0.001
A, n, %	0 (0)	22 (28.9)	
B, n, %	30 (40.0)	49 (64.5)	
E, n, %	45 (60.0)	5 (6.6)	

BNP, b-type natriuretic peptide; FEV1, forced expiratory volume in 1 second; FVC, forced vital capacity; GOLD, global initiative for chronic obstructive lung disease; hs-CRP, high-sensitivity C-reactive protein; RDW, red blood cell distribution width; WBC, white blood cell. P values were calculated by t-test or Mann-Whitney test where applicable. For data denoted by^a^, comparisons were performed using the t – test. For data marked by^b^, comparisons were carried out using the Chi – square test. Other comparisons were conducted using the Mann – Whitney test.

### Correlation analysis

The correlations between FEV1/predicted FEV1, FEV1/FVC and clinical and laboratory parameters were summarized in Table 2. We found that the RDW was inversely correlated with FEV1/predicted FEV1 (SRCC = −0.183, p=0.025) and FEV1/FVC (SRCC = −0.252, p=0.002). In addition, ABE group was also inversely correlated with FEV1/predicted FEV1 (SRCC = −0.792, p<0.001) and FEV1/FVC (SRCC = −0.781, p<0.001).

**Table 2:** Correlation analysis between various clinical and laboratory parameters with FEV1/predicted FEV1 and FEV1/FVC in COPD patients.

Variables	FEV1/predicted FEV1		FEV1/FVC	
	SRCC	p-Value	SRCC	p-Value
Age, years	−0.091	0.265	−0.149	0.068
Albumin, g/L	−0.117	0.155	−0.151	0.066
Cystatin C, mg/L	−0.114	0.162	−0.146	0.074
White blood cell count ( × 10^9^/L)	−0.038	0.643	−0.035	0.669
Eosinophil count ( × 10^9^/L)	0.093	0.254	0.022	0.789
Hemoglobin, g/L	−0.097	0.235	−0.078	0.341
Red blood cell distribution width, %	−0.183	0.025	−0.252	0.002
hs-CRP, mg/L	0.020	0.807	0.056	0.499
Fibrinogen, g/L	−0.066	0.421	−0.095	0.244
D-dimer, mg/L	−0.031	0.708	0.021	0.796
CD3, %	−0.078	0.341	−0.054	0.511
CD4, %	−0.057	0.490	−0.076	0.355
CD8, %	−0.088	0.282	−0.022	0.792
CD4/CD8	0.001	0.991	−0.049	0.551
Procalcitonin, ng/ml	−0.051	0.533	−0.0222	0.789
B-type natriuretic peptide, pg/ml	0.058	0.484	0.027	0.745
pH	0.089	0.276	0.105	0.200
ABE group	−0.792	<0.001	−0.781	<0.001

FEV1, forced expiratory volume in 1 second; FVC, forced vital capacity; hs-CRP, high-sensitivity C-reactive protein. The correlation coefficient ρ was calculated by Spearman’s correlation analyses.

### Univariable and multivariable analysis

**Figure 2:**
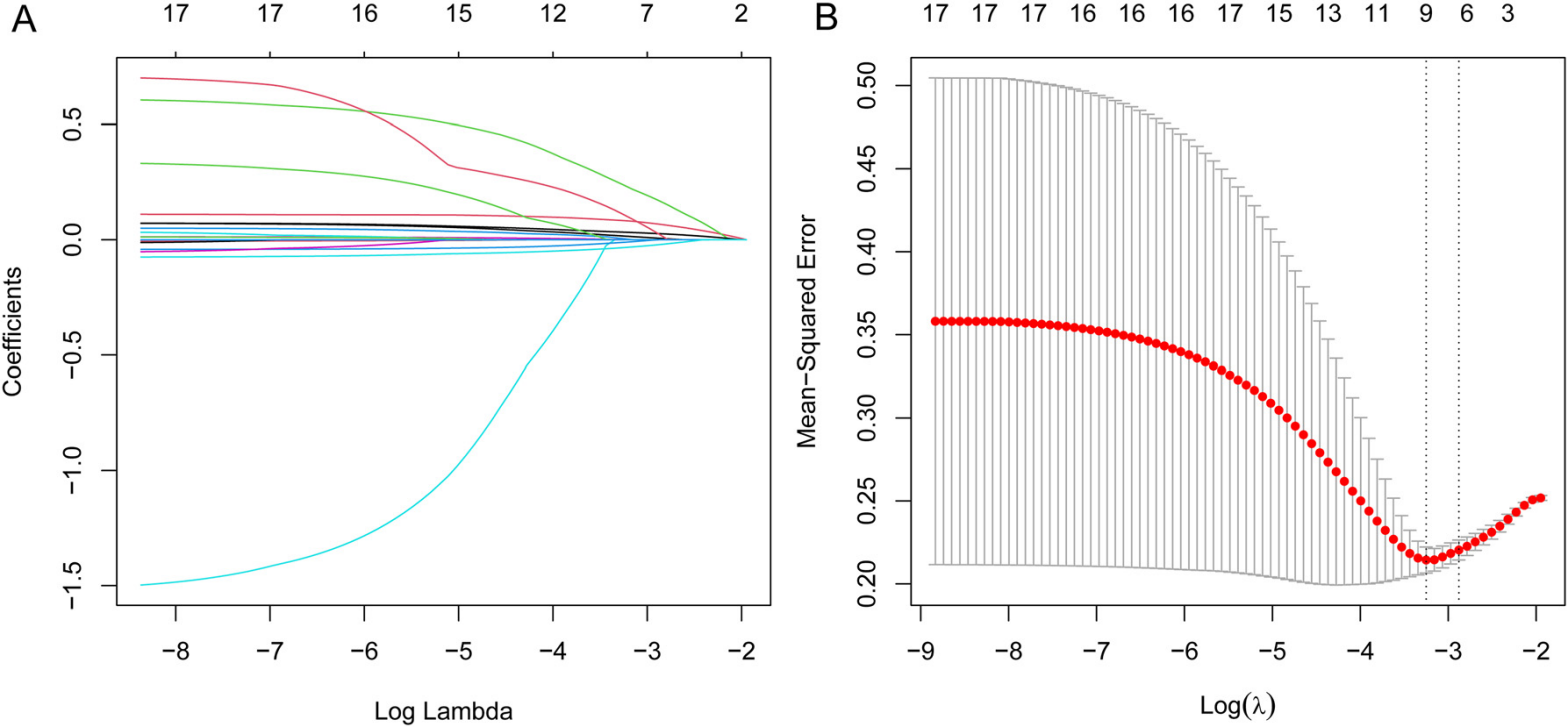
LASSO regression for variable selection in chronic obstructive pulmonary disease (COPD) with pulmonary hypertension. (A) Coeffïcient profïle plot of predictors; (B) cross-validation plot.

Due to complete separation in logistic regression caused by the absence of category A in the ABE classification within the COPD without PH group, and the potential for unstable parameter estimates from the extremely small sample size of category E (n=5) in COPD patients with PH, the ABE classification was excluded from the prediction model to ensure its applicability and robustness. The remaining 17 laboratory variables were subjected to LASSO regression for variable selection, yielding six selected variables (age, albumin, cystatin C, RDW, pH, and CD4/CD8 ratio) that were subsequently included in univariate and multivariate logistic regression analyses (Figure 2).

As presented in Table 3, univariable analysis indicated that serum albumin, cystatin C, RDW and pH were significantly related to PH in COPD (p<0.05). The multivariable-adjusted logistic regression showed that serum albumin (p<0.001), cystatin C (p=0.003), RDW (p=0.017), pH (p=0.018) and Age (p=0.024) were independent predictors for COPD patients with PH.

**Table 3:** Univariate and multivariate logistic regression for the identification of pulmonary hypertension in patients with chronic obstructive pulmonary disease using continuous variables.

Variables	Univariate analysis		Multivariate analysis	
	Or (95 % CI)	p-Value	Or (95 % CI)	p-Value
Albumin, g/L	1.126 (1.053, 1.210)	<0.001	1.157 (1.069, 1.251)	<0.001
Cystatin C, mg/L	1.428 (1.128, 1.895)	0.007	1.571 (1.172, 2.106)	0.003
RDW, %	1.064 (1.026, 1.109)	0.002	1.051 (1.009, 1.094)	0.017
pH (per 0.01)	0.940 (0.888, 0.991)	0.024	0.928 (0.873, 0.987)	0.018
CD4/CD8	1.269 (0.935, 1.756)	0.134	1.429 (0.980, 2.086)	0.064
Age	1.022 (0.995, 1.052)	0.126	1.050 (1.006, 1.095)	0.024

Among continuous variables, except for pH value, the OR, value of other variables represents the change in the risk of COPD, complicated with pulmonary hypertension for every one – unit increase. CI, confidence interval; OR, odds ratio; RDW, red blood cell distribution width.

### Nomogram construction

**Figure 3:**
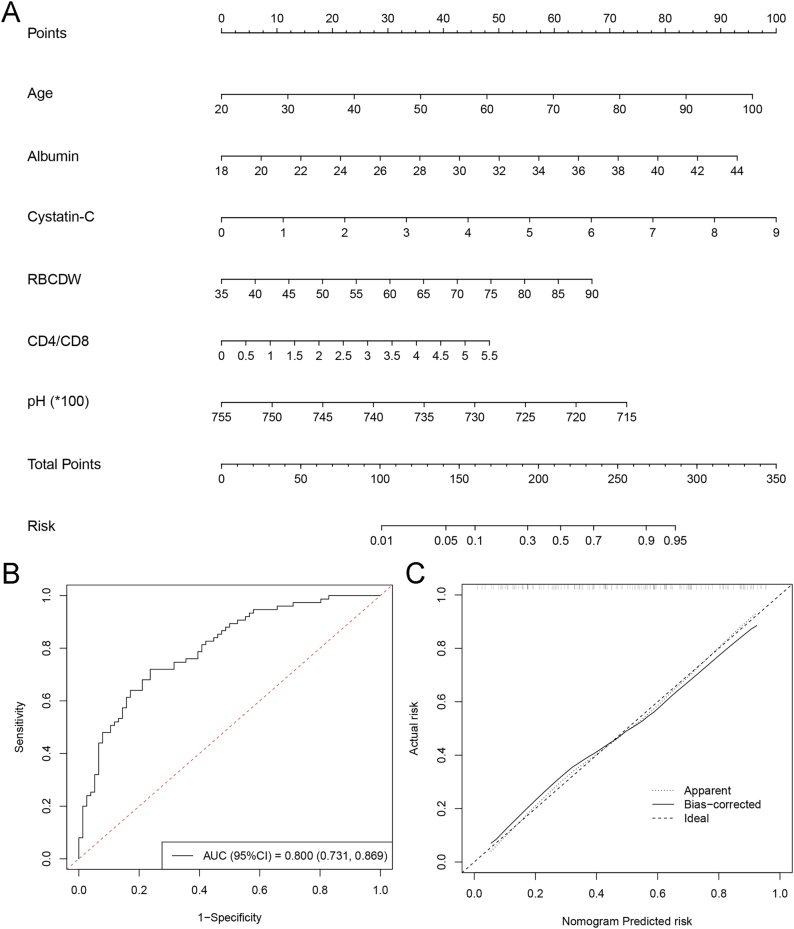
Construction and performance analysis of a nomogram for the prediction of the likelihood of pulmonary hypertension in patients with chronic obstructive pulmonary disease based on continuous variables. (A) The nomogram based on age, serum albumin, cystatin C, red blood cell distribution width (RDW), CD4/CD8, and pH. (B) receiver-operating characteristic curve for the nomogram. (C) calibration curve of the nomogram.

A nomogram for PH in COPD patients was thus constructed based on Age, serum albumin, cystatin C, RDW, CD4/CD8, and pH (Figure 3A). The total score for these 6 parameters ranged from 0 to 350 points, and the likelihood of PH ranged from 1 % to 95 % with a score within 101–286 points. The C-index for the nomogram was 0.8 (95 % confidence interval 0.731–0.869) (Figure 3B), and the Hosmer-Lemeshow test result for the calibration curve indicated good agreement between the observed PH risk and the nomogram’s predictions (Figure 3C).

## Discussion

In the present single-center retrospective study, we explored independent predictors for PH in COPD patients, and constructed a nomogram based on laboratory parameters of Age, serum albumin, cystatin C, RDW, CD4/CD8, and pH. This nomogram had good discriminatory capabilities and may be potentially clinically useful for the convenient prediction of PH during acute exacerbations in COPD patients.

Right heart catheterization remains the gold standard for diagnosing PH [17]. In this single-center retrospective study, the majority of patients were hospitalized for acute exacerbation of COPD, a factor that renders right heart catheterization not only impractical but often clinically inappropriate since right heart catheterization is an invasive procedure requiring arterial/venous puncture and fluoroscopic guidance, which increases the risk of complications in frail COPD patients. Besides, right heart catheterization costs approximately 5,000 RMB per procedure in China, which is not fully covered by basic medical insurance for most patients, creating a substantial financial barrier. In contrast, echocardiography costs only ∼200 RMB and is widely reimbursed, making it accessible even in resource-limited settings. Therefore, in our institution, echocardiography was chosen to estimate pulmonary artery systolic pressure primarily due to its clinical practicality and data availability. For the accuracy of PH diagnosis, prior studies have shown that echocardiographic estimates of pulmonary artery systolic pressure had a sensitivity and specificity of 90 and 70 %, respectively, for the diagnosis of PH [14]. To minimize biases, all echocardiograms were performed by experienced sonographers using the same equipment, reducing variability in measurements. Besides, PH were defined as estimated PASP≥40 mmHg, a threshold widely used in clinical and research settings for echocardiographic PH diagnosis in COPD [11]. However, early and accurate identification of PH in COPD patients by clinical parameters and routinely performed laboratory tests remained particularly helpful in resource-limited settings.

We identified serum albumin, cystatin C, RDW, pH, and age as potential predictors of PH in COPD. Notably, the observed positive association between elevated serum albumin levels and increased PH risk in COPD patients represents a paradoxical relationship requiring contextual interpretation. This finding contrasts with existing evidence: a meta-analysis of 16 studies (2,554 COPD patients, 2,055 non-COPD controls) reported significantly lower serum albumin concentrations in stable COPD patients versus controls [18]. Similarly, Snipelisky et al. demonstrated that pulmonary arterial hypertension (PAH; WHO Group 1) patients with hypoalbuminemia (serum albumin <33 g/L) exhibited reduced hemoglobin levels, higher hepatorenal dysfunction prevalence, and a 51.5 % lower mortality risk compared to those with albumin >33 g/L [19]. Conversely, another study found no statistically significant difference in albumin levels between COPD patients at low versus intermediate-to-high risk of PH (38.51 ± 5.34 g/L vs. 36.41 ± 5.15 g/L, p=0.466) [20]. These findings contrast with our results, which revealed significantly higher serum albumin levels in COPD patients with PH compared to those without PH. These discrepancies may be attributed to fundamental differences in the clinical characteristics of the studied populations. Our cohort specifically comprised COPD patients hospitalized for acute exacerbation, a condition characterized by systemic inflammation and oxidative stress that may transiently elevate serum albumin as a compensatory response to capillary leakage or hemodilution. Moreover, in COPD-PH, chronic hypoxia-induced endothelial dysfunction and vascular remodeling may alter albumin metabolism, wherein elevated albumin levels might reflect a maladaptive response to counteract oxidative stress or maintain oncotic pressure amid increased pulmonary vascular resistance. Given these complexities and contradictory evidence in stable COPD cohorts, this association should be interpreted as context-specific to acute exacerbation and not extrapolated to stable disease. Further prospective studies stratifying albumin levels by disease stability and PH etiology are warranted to elucidate these complex relationships.

Cystatin C is a non-glycosylated protein found in virtually all nucleated cells. In addition to serving as an endogenous biomarker of renal filtration independent of age, sex and muscle mass, cystatin C levels have also been associated with cardiac function and are predictive of cardiovascular mortality [21]. Furthermore, cystatin C level was found to be significantly elevated in COPD patients and negatively associated with lung function indicators, such as FEV1/predicted FEV1 and FEV1/FVC [22]. The present study demonstrated that cystatin C is an independent predictor of PH in COPD patients, which is consistent with the findings by Fenster’s group that cystatin C correlates with right ventricular systolic pressure, right ventricular end-systolic volume and right ventricular mass index in PAH (WHO Group 1) patients [23]. In another study, Duan reported that cystatin C is inversely correlated with tricuspid annular plane systolic excursion and that a cystatin C level >1.0 mg/dl predicts risk of mortality in PH patients [24].

RDW quantifies erythrocyte volume heterogeneity, with elevation indicating dysfunctional erythropoiesis, increased red cell destruction, or shortened red cell lifespan, all occurring in COPD patients with persistent hypoxia and hypercoagulability. Yang observed that COPD patients with PH had significantly higher RDW levels than those without, which aligns with our findings [25]. Moreover, we also noted that RDW is inversely correlated with FEV1/predicted FEV1 and FEV1/FVC, suggesting RDW is a sensitive biomarker for lung function. Pan et al. demonstrated that RDW remains inversely correlated with FEV1, FVC, and FEV1/FVC even after multivariate adjustments in the general population [26]. Arterial blood gas pH reflects the degree of hypoxia and is widely used in clinical practice. Previous studies have consistently shown that lower pH values correlate with COPD severity and both short- and long-term mortality [27], 28]. In fact, sustained hypercapnia and acidosis have been shown to increase pulmonary vasoconstriction, potentially contributing to PH development [29]. Although RDW and pH were statistically significant in univariate analysis, their independent contributions to COPD complicated by PH may not have met the inclusion criteria for the multivariate model.

Nomograms are tools for individualized prediction of clinical event probabilities by integrating diverse variables, and have been widely and successfully used in various medical fields [30], 31]. Due to the limitations of right heart catheterization, several studies have developed nomogram models to predict disease progression in COPD. For example, Wang et al. created a nomogram incorporating GOLD stage, emphysema, PaCO_2_, NT-pro-BNP, red blood cell distribution width-standard deviation (RDW-SD), and neutrophil-to-lymphocyte ratio (NLR), achieving an area under the curve (AUC) of 0.770 [20]. Similarly, Wu et al. developed a nomogram for predicting acute heart failure in COPD patients with an AUC of 0.8195 [32]. In this study, we constructed a nomogram to assess the probability of pulmonary hypertension (PH) in COPD patients using readily available laboratory parameters. Our model demonstrated good discriminative ability and clinical accuracy. Notably, all parameters included in our nomogram are routinely accessible in clinical practice. While our study primarily identified predictors of PH in COPD patients, the progression of PH to right heart failure remains a critical concern. A recent study developed a nomogram to predict acute heart failure risk in COPD patients, emphasizing the importance of early intervention to prevent decompensation [32]. Our nomogram, which includes age, serum albumin, cystatin C, RDW, CD4/CD8 ratio, and pH, may serve as a complementary tool in this context.

The strength of this study lies in addressing the need for a predictive model to enable early and accurate identification of pulmonary hypertension (PH) in COPD patients. This nomogram is specifically designed for clinical scenarios where echocardiography is unavailable, delayed, or contraindicated – such as in primary care settings lacking equipment, during periods of high demand, or for patients unwilling or unable to tolerate echocardiography due to severe illness. While echocardiography remains a valuable non-invasive tool, its clinical utility is often limited by practical constraints that our blood-based nomogram helps overcome. In many healthcare settings, echocardiography equipment or experienced sonographers are not readily accessible. Even in tertiary hospitals, urgent echocardiography during COPD exacerbations is frequently delayed due to high demand, with wait times exceeding 24–48 hours. In contrast, serum albumin, cystatin C, and ABE group measurements are routinely obtained upon admission for COPD patients, typically available within hours of hospitalization. This enables immediate risk stratification when echocardiography is unavailable or delayed. Moreover, echocardiography, though informative, is not without cost or risk. Routine universal echocardiography for all COPD patients is neither practical nor cost-effective. Our nomograms provide mechanistic insights while addressing accessibility gaps, enabling early, non-invasive PH identification in COPD patients using routine laboratory parameters. By bridging the diagnostic delay before echocardiography, these user-friendly tools guide clinical decision-making in resource-limited settings where invasive testing is unavailable. Clinicians sum mapped parameter scores to derive the predicted probability, stratify patients into risk groups, and facilitate timely intervention.

Nonetheless, the results of this study should be interpreted with caution with the recognition of the following limitations. First, this is a single-center study with relatively limited sample size, rendering generalization and external extrapolation difficult. Second, the study design is retrospective, and thus study population may be potentially subject to selection bias. Besides, due to the retrospective nature of our study, other parameters that might influence pulmonary hemodynamics, such as home oxygen therapy were not recorded. Third, although echocardiography has been frequently used clinically for the diagnosis of PH, it has inherent limitations including operator dependency, suboptimal acoustic windows due to lung hyperinflation in COPD patients, and potential inaccuracies in pressure estimation. Technical limitations such as hyperinflation, poor acoustic windows, and inadequate tricuspid regurgitation jet visualization are particularly pronounced in COPD populations and can substantially reduce the diagnostic accuracy of transthoracic echocardiography, potentially leading to underestimation or overestimation of pulmonary artery pressures. Furthermore, right heart strain may begin early even at mild PH stages [33], emphasizing the importance of comprehensive right ventricular function assessment beyond PASP estimation alone [34]. Most importantly, acute exacerbations of COPD can cause transient increases in pulmonary artery pressure through mechanisms such as hypoxic vasoconstriction, dynamic changes in intrathoracic pressure, and altered volume status. Fourth, it is important to note that exacerbations, particularly those associated with hypoxia, can transiently elevate pulmonary artery pressures, potentially leading to overestimation of the prevalence of PH. Ideally, multi-center prospective studies with the right ventricular catheterization serving as the gold standard can be performed to conclusively elucidate the determinants of PH in COPD patients. Fifth, due to complete separation in logistic regression caused by the absence of category A in the ABE classification within the COPD without PH group and the extremely small sample size of category E (n=5) in COPD patients with PH, we were unable to incorporate ABE classification into the prediction model or perform subgroup analyses stratified by ABE groups. This limitation prevents assessment of nomogram performance across different ABE categories and restricts our ability to determine whether the model’s predictive accuracy varies by disease severity classification. Future multi-center prospective studies with larger, more balanced cohorts across all ABE categories are needed to develop and validate ABE-stratified prediction models for pulmonary hypertension in COPD patients. Furthermore, the observed prevalence of PH (approximately 49.7 %) in our cohort was higher than that reported in some large-scale database studies. This discrepancy may be attributed to several factors inherent to our study design. Firstly, as a single-center retrospective study aiming to identify predictors, our study population comprised hospitalized COPD patients who underwent echocardiography, rather than all consecutively admitted patients. This selective inclusion based on data completeness might have led to an over-representation of patients with more severe conditions or clinical suspicion of PH, thus introducing selection bias and potentially overestimating the PH prevalence. Secondly, acute exacerbations can transiently elevate pulmonary artery pressure. Therefore, a proportion of PH cases identified in this study during the acute exacerbation phase might represent reversible PH rather than fixed chronic pulmonary vascular disease. Consequently, the nomogram developed in this study is primarily intended for risk stratification during acute exacerbations and its applicability to stable COPD patients or for predicting fixed PH requires further validation.

## Conclusions

In summary, this single-center retrospective analysis generated a nomogram based on age, serum albumin, cystatin C, RDW, CD4/CD8, and pH for assessing the likelihood of PH during acute exacerbations of COPD. Additional prospective multi-center studies are needed to validate our findings and to assess the potential utility of the nomograms constructed.
